# HR+/HER2– breast cancer brain metastases treated with definitive local therapy: a focus on endocrine sensitivity and incidence of leptomeningeal disease

**DOI:** 10.1093/noajnl/vdag048

**Published:** 2026-02-26

**Authors:** Jie Wei Zhu, Arjun Sahgal, Veronika Moravan, Katarzyna J Jerzak

**Affiliations:** Department of Medicine, University of Toronto (J.W.Z.); Department of Medicine, Sunnybrook Odette Cancer Centre, University of Toronto (J.W.Z., K.J.J.); Department of Radiation Oncology, Sunnybrook Odette Cancer Centre, University of Toronto (A.S.); VM stats, Toronto, ON, Canada (V.M.); Department of Medicine, Sunnybrook Odette Cancer Centre, University of Toronto (J.W.Z., K.J.J.)

**Keywords:** brain metastases, breast cancer, endocrine resistance, leptomeningeal disease, systemic treatment

## Abstract

**Background:**

Brain metastases (BrM) among patients with hormone receptor-positive (HR+)/human epidermal growth factor receptor 2-negative (HER2–) metastatic breast cancer (mBC) are understudied. We sought to understand the endocrine sensitivity of patients with HR+/HER2– BrM and determine the associated risk of leptomeningeal metastatic disease (LMD).

**Methods:**

We conducted a retrospective cohort study of 204 consecutive patients (≥18 yr of age) with HR+/HER– mBC who were treated for BrM at the Sunnybrook Odette Cancer Centre between 2008 and 2018. We used descriptive statistics to summarize patient and treatment characteristics and Kaplan-Meier analyses for survival outcomes. The log-rank test was used to compare outcomes of patients with endocrine-sensitive versus endocrine-resistant disease.

**Results:**

Among 204 patients with HR+/HER2– BrM, the median age at BrM diagnosis was 56 years (range, 48-64), and the median time between diagnosis of mBC to development of BrM was 15 months (interquartile range [IQR], 3-36). At the time of BrM development, 160 patients (78.4%) had endocrine-resistant disease, and 28 patients (13.7%) had endocrine-sensitive disease; endocrine sensitivity of the remaining 16 patients (7.8%) was unknown. In total, 58 patients (28.4 %) developed LMD with a median time from BrM to LMD of 10.8 months (IQR, 3.1-14.8). Patients with endocrine-sensitive disease at the time of BrM diagnosis had a longer brain-specific progression-free survival (7 mo versus 5 months, *P* = .004) and overall survival (24 months versus 5 mo, *P*  = .002) compared with those with endocrine resistance.

**Conclusions:**

Most patients with HR+/HER2– BrM have endocrine-resistant disease, with an unexpectedly high likelihood of developing LMD.

Key points

- Patients with HR+/HER2– mBC develop BrM after a median of 2 lines of systemic therapy and a median of 15 months after diagnosis with mBC
- Most patients with HR+/HER2– BrM (78.4%) had endocrine-resistant disease, associated with a median survival of 9 months.
- ∼1 in 4 patients with HR+/HER2– BrM developed LMD.

Importance of Study

This study offers novel insights regarding the pattern of development of BrM among patients with HR+/HER2– mBC and identifies a high prevalence of endocrine resistance (78.4%) at the time of BrM diagnosis, which was associated with a median OS of only 5 months. In contrast, patients with endocrine-sensitive disease had a much longer median OS of 24 months, suggesting that some patients have potential to benefit meaningfully from endocrine-based strategies. In addition, an unexpectedly high incidence of leptomeningeal metastatic disease was identified (28.4%) in this patient population. Our study is limited by a relatively small sample size and the low proportion of patients who received CDK4/6 inhibitors during the study period. Nevertheless, given that BrM are more common than previously recognized among patients with HR+/HER2– MBC and are associated with a poor prognosis, novel treatment strategies for this patient population are urgently required.

Breast cancer (BC) is the most common cancer in women worldwide, with 2.3 million new cases and 685,000 breast cancer-related deaths in 2020.^1^ High recurrence rates and risk of developing of distant metastases contribute to high mortality rates. In fact, at least 1 in 5 women with early BC will develop incurable metastatic disease,^2^ with hormone receptor-positive (HR+)/human epidermal growth factor receptor 2-negative (HER2–) BC accounting for 65-70% of cases.^3^

Although brain metastases (BrM) are not as common among patients with HR+/HER2– breast cancer as those with HER2+ or triple negative disease, ∼14% will develop BrM in their lifetime.^4–6^ Further, given the high prevalence of HR+/HER2– BC, this subtype represents approximately one-third of all breast cancer BrM.^7^^,^^8^ Currently, there are no standardized guidelines for the systemic treatment of BrM among patients with HR+/HER2– metastatic BC (mBC) due to a lack of prospective trials specific to this population. Treatment options generally include surgery and/or radiation therapy.^9^

The standard of care first-line systemic treatment for patients with HR+/HER2– mBC is currently endocrine therapy (ET) with a cyclin-dependent kinases 4 and 6 inhibitor (CDK4/6i).^10^ While limited data inform the efficacy of CDK4/6i in the brain,^11^ a single arm phase II study of abemaciclib (± ET) demonstrated an intracranial clinical benefit rate (duration of clinical benefit ≥6 mo) of 24% in 58 patients with HR+/HER2– new or not previously irradiated and measurable BrM (*n* = 58, 100%); the median number of prior lines of systemic therapy was 3 (range 1-10).^12^ While some data suggest that BrM are more common among patients whose BC harbors an activation of the phosphoinositide 3 kinase (PI3K)/Akt/mammalian target of rapamycin (mTOR) pathway,^13^ relevant randomized trials evaluating the efficacy of PI3K inhibitors often exclude patients with BrM and lack central nervous system (CNS)-specific outcomes.^14^^,^^15^ Although data regarding CNS-specific efficacy of PI3K inhibitors in patients with HR+/HER2– BrM are limited,^16^ an ongoing phase II trial is evaluating the activity of targeted agents including CDK4/6i and PI3K inhibitors in patients with progressive brain metastases from breast, lung and other primary cancers (NCT03994796).

To help inform the design of future clinical trials for patients with HR+/HER2– mBC and BrM, we sought to assess the treatment patterns and outcomes of this patient population at our institution.^8^ Our primary goal was to establish the proportion of patients whose disease was considered endocrine sensitive (and thereby could be considered for novel endocrine-based trial interventions) at the time of BrM diagnosis.

## Methods

### Study Design and Population

This is a single-institution retrospective study of patients (≥18 yr of age) identified consecutively from a pre-existing database of patients with a diagnosis of HR+/HER– mBC treated between 2008 and 2018 for BrM with initial surgery, and/or whole-brain radiotherapy (WBRT) and/or stereotactic radiosurgery (SRS), at the Sunnybrook Odette Cancer Centre, Toronto, Canada. Males, patients with a history of other primary malignancies, and those with an uncertain date of breast cancer brain metastases diagnosis were excluded. Histological confirmation of breast cancer was required for all patients. However, central repeat testing of BrM tissue for biomarker status was not routinely performed or required for inclusion. HER2 status was defined on the basis of IHC and ISH reported at the time of initial pathology review; in patients with more than one biopsy with HER2 testing available, HER2 status was assigned on the basis of the biopsy with the higher HER2 status. This study was approved by our institutional ethics review board. Due to the retrospective nature of the study, the Research Ethics Board of Sunnybrook Health Sciences Centre waived the need of obtaining informed consent.

### Study Outcomes

The main goal of this study was to determine the proportion of patients with HR+/HER2– mBC whose disease was considered endocrine sensitive versus resistant at the time of BrM development. The diagnosis of BrM was established based on imaging (contrast-enhanced MRI or CT head) and supportive clinical features. The date of BrM diagnosis was defined as the time of first radiographic evidence of BrM.

According to international consensus guidelines, we defined endocrine resistance as relapse while on adjuvant ET, within 12 months of completing adjuvant ET, or disease progression while on first-line ET for mBC; patients were considered to have endocrine sensitive disease if they never received ET for early breast cancer, experienced relapse ≥12 months after completing adjuvant ET, or if they were diagnosed with de novo mBC.^17^ To determine extracranial disease status at the time of BrM development, we used the results of computerized tomography (CT), bone scans and/or MRI findings, and clinical notes from patient electronic medical records within 4 weeks of the date of local BrM therapy. Patients with systemic disease progression within 4 weeks of local therapy were classified as having “progressing” extracranial disease; the remaining patients had “stable” extracranial disease.

Among patients with endocrine resistant disease, we described the time from initiation of chemotherapy for mBC to the development of BrM. Among patients with endocrine-sensitive disease, we described the time from initiation of ET in the metastatic setting to the development of BrM. In addition, we assessed factors that were independently associated with brain-specific progression-free survival (bsPFS), overall progression-free survival (PFS) and overall survival (OS) in this patient population. bsPFS was defined as time from BrM diagnosis until the date of radiographic disease progression in the brain (patients with systemic disease progression were not censored by definition). PFS was defined as the time from initial diagnosis of mBC to time of either progression in the brain, extracranial progression, or death. OS was defined as the time from BrM diagnosis to the date of death due to any cause. Dates of local treatment to the brain were used as surrogates for progression in cases where the date of progression was not documented. Clinical outcomes of interest were compared between patients with endocrine-sensitive versus endocrine-resistant disease.

### Statistical Methods

Clinical and treatment characteristics are described with median and interquartile range (IQR) for continuous variables, or frequencies and percentages for categorical variables. Kaplan-Meier curves were generated for PFS, bsPFS and OS; log-rank tests were used to compare curves. Cox proportional hazards regression was used to estimate hazard ratios (HR) and identify factors associated with PFS, bsPFS and OS. Univariable analyses were performed for all covariates. Bayesian Information Criteria determined the best models from all subsets of covariates. The proportional-hazards assumption was appraised by visually inspecting graphs of Schoenfeld residuals and testing their independence over time. All analyses were performed using R software version 4.4.2.^18^ All *P* values were 2-sided, and statistical significance was determined using the traditional *P*-value of < .05.

## Results

### Patient Characteristics

We included a total of 204 patients with HR+/HER2– breast cancer and BrM in this study (**Table 1**). The median age at BrM diagnosis was 56 years (range, 48-64), and the median time from diagnosis of MBC to the diagnosis of BrM was 15 months (IQR, 3-36). The median time from initial breast cancer diagnosis to the development of mBC was 38 months (IQR, 13-74) and median time to BrM diagnosis was 67 months (IQR, 32-109). Almost all (98.5%) patients had extracranial disease, most commonly with metastases to bone (84.3%), liver (64.7%), and lung (58.8%); only 24 patients (11.8%) had bone metastases as their only extra-cranial site of disease. At the time of BrM diagnosis, 132 patients (64.7%) had concurrent extracranial disease progression, while 69 patients (33.8%) had stable extracranial disease and 3 patients (1.5%) had unknown extracranial disease status.

**Table 1.** Baseline clinical and treatment characteristics stratified by endocrine sensitivity

	All patients	Endocrine resistant	Endocrine sensitive	Undetermined
	*N* = 204 (%)	*N* = 160 (%)	*N* = 28 (%)	*N* = 16 (%)
**Age at BrM diagnosis**				
Mean (SD)	57 (11.7)	56 (11)	60.5 (12.5)	61.4 (15.4)
Median (IQR)	56 (48-64.2)	55 (48-63)	62.5 (49.2-68.2)	62.5 (49.2-74.2)
Min, Max	31, 93	31, 93	42, 88	39, 88
**Age at BrM diagnosis**				
<50 years	59 (28.9)	48 (30)	7 (25)	4 (25)
≥50 years	145 (71.1)	112 (70)	21 (75)	12 (75)
**Months from EBC to BrM diagnosis**				
Mean (SD)	77 (59)	72.5 (55.1)	114 (68.6)	57.5 (56.3)
Median (IQR)	67 (31.5-108.5)	62 (30.5-103)	101 (76.2-135.8)	27 (18.5-94.5)
Min, Max	0, 318	0, 309	1, 318	5, 183
**Months from EBC to MBC diagnosis**				
Mean (SD)	50.2 (46.7)	48.4 (43.9)	57 (57.4)	55.9 (55.3)
Median (IQR)	38 (13-74)	38 (13-72)	49 (10.8-74.2)	28 (17-86.5)
Min, Max	0, 256	0, 174	0, 256	5, 183
**Months from MBC to BrM diagnosis**				
Mean (SD)	26.5 (30.9)	23.2 (24.4)	57 (47.4)	3.9 (11.4)
Median (IQR)	15.5 (3.2, 36)	15 (6, 35)	51.5 (24.8, 78.5)	0 (0, 0)
Min, Max	0, 180	0, 147	0, 180	0, 44
**Systemic treatment lines for MBC prior to BrM**				
None	40 (19.6)	31 (19.7)	4 (14.3)	5 (55.6)
1 line	45 (22.1)	41 (26.1)	1 (3.6)	3 (33.3)
2-3 lines	66 (32.4)	51 (32.5)	14 (50)	1 (11.1)
4-11 lines	43 (21.1)	34 (21.7)	9 (32.1)	0 (0)
Not known	10 (4.9)	3 (NA)	0 (NA)	7 (NA)
**Systemic treatment lines for BrM **				
None	30 (14.7)	24 (16)	4 (16.7)	2 (25)
1 line	75 (36.8)	61 (40.7)	9 (37.5)	5 (62.5)
2-3 lines	55 (27)	49 (32.7)	5 (20.8)	1 (12.5)
4-9 lines	22 (10.8)	16 (10.7)	6 (25)	0 (0)
Not known	22 (10.8)	10 (NA)	4 (NA)	8 (NA)
**Neurological symptoms at BrM diagnosis**				
Yes	158 (77.5)	123 (78.3)	21 (80.8)	14 (87.5)
No	41 (20.1)	34 (21.7)	5 (19.2)	2 (12.5)
Unknown	5 (2.5)	3 (NA)	2 (NA)	0 (NA)
**Leptomeningeal disease (LMD)** ^a^				
Yes	58 (28.4)	45 (28.1)	8 (28.6)	5 (31.2)
No	146 (71.6)	115 (71.9)	20 (71.4)	11 (68.8)
**No. of BrM lesions**				
Single metastasis	29 (14.2)	17 (16)	7 (41.2)	5 (41.7)
Multiple metastases	106 (52)	89 (84)	10 (58.8)	7 (58.3)
Not known	69 (33.8)	54 (NA)	11 (NA)	4 (NA)
**Extracranial disease status at BrM diagnosis**				
Stable	69 (33.8)	46 (28.9)	11 (40.7)	12 (80)
Progressing	132 (64.7)	113 (71.1)	16 (59.3)	3 (20)
Not known	3 (1.5)	1 (NA)	1 (NA)	1 (NA)
**Lung metastasis**				
Yes	120 (58.8)	102 (65.4)	17 (63)	1 (6.2)
No	79 (38.7)	54 (34.6)	10 (37)	15 (93.8)
Unknown	5 (2.5)	4 (NA)	1 (NA)	0 (NA)
**Liver metastasis**				
Yes	132 (64.7)	115 (71.9)	15 (55.6)	2 (12.5)
No	71 (34.8)	45 (28.1)	12 (44.4)	14 (87.5)
Unknown	1 (0.5)	0 (NA)	1 (NA)	0 (NA)
**Bone metastasis**				
Yes	172 (84.3)	146 (92.4)	24 (85.7)	2 (12.5)
No	30 (14.7)	12 (7.6)	4 (14.3)	14 (87.5)
Unknown	2 (1)	2 (NA)	0 (NA)	0 (NA)
**Breast cancer histology**				
Ductal	149 (73)	113 (85.6)	23 (92)	13 (100)
Lobular	9 (4.4)	9 (6.8)	0 (0)	0 (0)
Mixed	12 (5.9)	10 (7.6)	2 (8)	0 (0)
Unknown	34 (16.7)	28 (NA)	3 (NA)	3 (NA)
**Surgery for BrM**				
Yes	21 (10.3)	11 (6.9)	6 (21.4)	4 (25)
No	183 (89.7)	149 (93.1)	22 (78.6)	12 (75)
**Radiation for BrM**				
Whole-Brain Radiation Therapy (WBRT) only	145 (71.1)	118 (73.8)	17 (60.7)	10 (62.5)
Stereotactic Radiosurgery (SRS) only	31 (15.2)	22 (13.8)	7 (25)	2 (12.5)
Both	18 (8.8)	13 (8.1)	3 (10.7)	2 (12.5)
None	10 (4.9)	7 (4.4)	1 (3.6)	2 (12.5)
**Treatment prior to metastases**				
Tamoxifen	120 (58.8)	96 (60)	18 (64.3)	6 (37.5)
Aromatase inhibitor	42 (20.6)	30 (18.8)	4 (14.3)	8 (50)
Chemotherapy	10 (4.9)	7 (4.4)	1 (3.6)	2 (12.5)
Not applicable-concurrently diagnosed with metastatic disease	25 (12.3)	20 (12.5)	5 (17.9)	0 (0)
Other (declined treatment, only radiation, unknown)	7 (3.5)	7 (4.4)	0 (0)	0 (0)
**First systemic treatment in metastatic setting**				
Endocrine	144 (70.6)	111 (69.4)	25 (89.3)	8 (50)
Chemotherapy	49 (24.0)	46 (28.7)	3 (10.7)	0 (0)
Unknown	11 (5.4)	3 (1.9)	0 (0)	8 (50)
**Systemic treatment after BrM diagnosis** ^b^				
Endocrine only	34 (16.7)	26 (16.2)	3 (10.7)	5 (31.2)
Chemotherapy only	77 (37.7)	64 (40)	13 (46.4)	0 (0)
Both chemotherapy and endocrine therapy	42 (20.6)	36 (22.5)	5 (17.9)	1 (6.2)
No systemic treatment	30 (14.7)	24 (15)	4 (14.3)	2 (12.5)
Unknown	21 (10.3)	10 (6.2)	3 (10.7)	8 (50)
**Months from initial chemotherapy for MBC to BrM^c^**				
Mean (SD)	11.5 (16.1)	11.7 (15.4)	16.9 (21.2)	0 (0)
Median (IQR)	4 (0-18)	6 (0-18.2)	5.5 (0-32.2)	0 (0-0)
**Timing of initial chemotherapy for MBC**				
≥13 Months before BrM	65 (31.9)	53 (33.1)	12 (42.9)	0 (0)
2-12 months before BrM	50 (24.5)	47 (29.4)	3 (10.7)	0 (0)
1 month before BrM or later	43 (21.1)	35 (21.9)	7 (25.0)	1 (6.2)
No chemo or no date for chemo	46 (22.5)	25 (15.7)	6 (21.4)	15 (93.8)

a LMD diagnosed concurrently at the time of BrM or following BrM.

b The discrepancy between 34 patients who received endocrine therapy after BrM diagnosis despite 28 patients having endocrine-sensitive disease is likely explained by the fact that some patients with endocrine-resistant disease may not have been fit for more aggressive therapies (ie chemotherapy) when BrM were diagnosed.

c Zero if no chemotherapy prior to BrM.

In our study population, 160 patients (78.4%) had endocrine resistant and 28 patients (13.7%) had endocrine sensitive disease; endocrine sensitivity versus resistance could not be adjudicated for 16 patients (7.8%) due to limited available data regarding their systemic treatments. In terms of local therapy, 21 patients (10.3%) had surgery, 145 patients (71.1%) had WBRT, 31 patients (15.2%) had SRS, 18 patients (8.8%) received both WBRT and SRS, and 10 patients (4.9%) were not treated with radiation.

### Leptomeningeal Disease

Among all patients in our cohort, 58 patients (28.4%) had leptomeningeal disease (LMD), and their median OS was 5.3 months (95% CI, 3.4-9.0). Among the 58 patients who developed LMD in our cohort, 49 (84.5%) patients had clinical symptoms, and 57 (98.3%) patients had MRI brain imaging. In total, 42 (72.4%) patients underwent MRI spine imaging and 15 (25.9%) patients did not; for 1 patient (1.7%) who was referred from an outside center, we could not confidently determine whether or not an MRI spine had been performed. Results of CSF analyses are only available for 2 (3.4%) patients; however, it is possible that more patients underwent CSF sampling at peripheral hospitals prior to referral to our tertiary care centre for the purpose of brain radiotherapy. 72.4% of patients were diagnosed with LMD concurrently with BrM (defined as within 2 months of BrM diagnosis). 27.6% of patients developed LMD following a diagnosis of BrM, with a median time from BrM to LMD of 10.8 months (IQR 3.1-14.8). Among patients with LMD, the most common histologic subtype was ductal carcinoma in 41 patients (83.7%), lobular carcinoma in 3 patients (6.1%), mixed carcinoma in 5 patients (10.2%) and unknown in 9 patients (15.5%). The median OS for patients with endocrine-sensitive disease and LMD was 11.8 months (95% CI, 1.4-NA) versus 5.3 months (95% CI, 3.1-9) for those with endocrine-resistant disease (*P* = .20).

### Systemic Treatment Patterns

The most common adjuvant ET for patients at the time of early-stage breast cancer was tamoxifen (58.8%), followed by an aromatase inhibitor (20.6%) and chemotherapy (4.9%). About 12.3% of patients were diagnosed with de novo metastatic disease, and treatment information was unavailable for 3.5% of patients. At the time of mBC diagnosis, 70.6% of patients received ET and 24.0% were treated with chemotherapy in the first-line setting; type of systemic therapy was not available for 5.4% of patients (Table 1). Only 12.3% of patients received a CDK4/6 inhibitor, the most common agent being palbociclib (10.3%).

The majority of patients developed BrM after mBC diagnosis (*n* = 165, 80.9%), while 39 (19.1%) were diagnosed with BrM concurrently with extracranial metastases. Most patients (54.4%) received 1 to 3 lines of systemic therapy, and *n* = 43 (42.4%) received 4 or more lines of systemic therapy prior to their diagnosis of BrM. In the metastatic setting prior to BrM diagnosis, the most common ET included aromatase inhibitors (60.8%), followed by tamoxifen (20.6%) and fulvestrant (19.0%), while the most common chemotherapy agents were capecitabine (37.1%), followed by paclitaxel (24.7%) and vinorelbine (10.8%). Following BrM diagnosis, most patients received chemotherapy (46.1%), while 19.6% initially received ET and subsequently switched to chemotherapy after a median of 8 months (IQR, 1-25 mo). About 16.7% received ET without a subsequent switch to chemotherapy, 14.7% did not receive any systemic therapy, and 10.3% of patients had unknown treatment status. Among those treated with chemotherapy prior to a BrM diagnosis, the median time between initiation of chemotherapy and the development of BrM was 4 months (IQR, 0-18).

### Survival Outcomes

**Figure 1.**
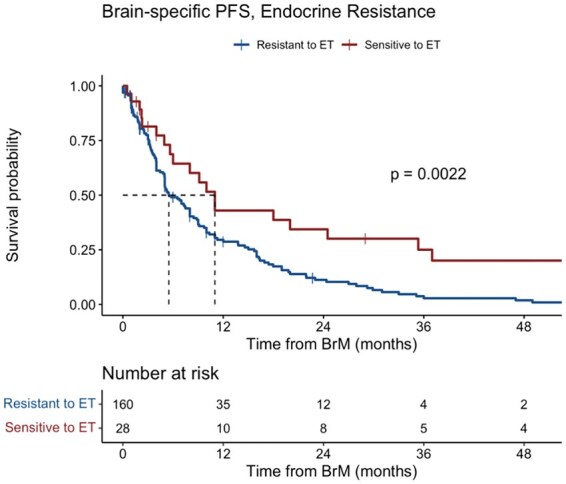
Kaplan Meier plot of brain-specific progression-free survival for endocrine resistance versus endocrine sensitive.

The median bsPFS was 7 mo (95% CI, 5.2-9). Multivariate analysis demonstrated that increasing age at BrM diagnosis (HR, 1.19; 95% CI, 1.10-1.50; *P* = .01) and progressive extracranial disease (HR, 1.53; 95% CI, 1.09-2.13; *P* = .01) at the time of BrM diagnosis were prognostic for shorter bsPFS (**Table 2**). The median bsPFS for patients with endocrine-resistant disease was significantly shorter at 5.5 months (95% CI, 5-8) compared with 11 months (95% CI, 6-37) for patients with endocrine sensitive disease (*P* = .002) (**Figure 1**).

**Table 2.** Factors associated with brain-specific progression-free survival in HR+/HER2– BrM

	Univariable analysis		Multivariable analysis	
	Hazard ratio (95% CI)	*P* value	Hazard ratio (95% CI)	*P* value
**Breast cancer histology**				
Ductal (ref)	1			
Lobular	1.29 (0.60-2.79)	.5124		
Mixed	1.12 (0.57-2.20)	.7522		
**Age at MBC^a^**	1.20 (1.06-1.37)	.0056	1.20 (1.05-1.38)	.0088
**Months from MBC to BrM (ln)** ^b^	1.08 (1-1.18)	.053		
**Endocrine resistance at 12 months**				
No (ref)	1			
Yes	2.15 (1.32-3.53)	.0023	1.98 (1.2-3.28)	.0078
Undetermined	1.73 (0.79-3.77)	.170	1.95 (0.86-4.4)	.1075
**Chemotherapy in MBC prior to BrM**				
No (ref)	1			
Yes	1.50 (1.09-2.08)	.0138		
**Systemic treatment lines for MBC prior to BrM**				
None	1			
1 line	1.27 (0.79-2.04)	.3215		
2 or more lines	1.74 (1.16-2.62)	.0074		
**First systemic treatment line for BrM**				
Chemotherapy (ref)	1			
Other systemic treatment	1.04 (0.72-1.49)	.8444		
None	3.23 (1.97-5.28)	<.0001		
**Fulvestrant at BrM**				
No (ref)	1			
Yes	0.40 (0.20-0.79)	.008		
**Neurological symptoms at BrM diagnosis**				
No symptoms (ref)	1			
Symptoms	1.20 (0.82-1.76)	.3394		
**No. of BrM lesions**				
Single metastasis	1			
>1 metastasis	1.36 (0.86-2.16)	.194		
**EC progression at BrM diagnosis**				
Stable (ref)	1		1	
Progressing	1.51 (1.08-2.11)	.0159	1.48 (1.04-2.11)	.0312
**Leptomeningeal Disease (LMD)**				
No LMD (ref)	1			
LMD	1.24 (0.88-1.75)	.2168		
**Surgery for BrM**				
No (ref)	1			
Yes	0.57 (0.35-0.93)	.0234		
**Stereotactic Radiosurgery (SRS) for BrM**				
No (ref)	1			
Yes	0.82 (0.53-1.25)	.346		

a Age at MBC, continuous, centered at 50, 10 yr increments.

b Natural logarithm transformation of months from MBC to BrM.

**Figure 2.**
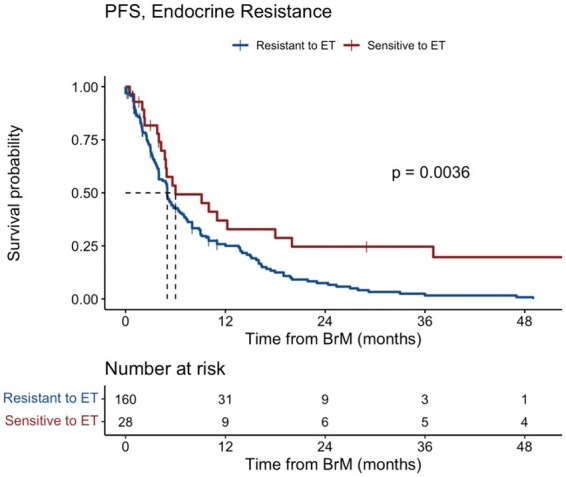
Kaplan-Meier plot of progression-free survival for endocrine-resistant versus endocrine-sensitive disease.

The median PFS in the overall cohort was 5.2 months (95% CI, 4.9-7). On multivariate analysis, increasing age was associated with significantly shorter PFS (HR, 1.16; 95% CI, 1.02-1.39; *P* = .03) while receiving surgery for BrM was associated with significantly longer PFS (HR, 0.54; 95% CI, 0.33-0.88; *P* = .01). The median PFS for patients with endocrine-resistant disease was 5 months (95% CI, 4-6.5) as compared with 6 months (95% CI: 4.7—20) for those with endocrine-sensitive disease (*P* = .004) (**Figure 2**).

**Figure 3.**
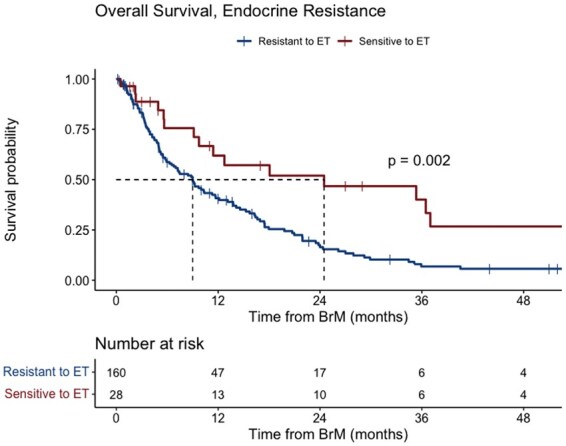
Kaplan-Meier plot of brain-specific overall survival for endocrine resistance versus endocrine sensitive.

The median OS of patients in our cohort was 10 months (95% CI, 8.5-13.4). On multivariate analysis, receiving chemotherapy prior to BrM (HR, 1.45; 95% CI, 1.01-2.08; *P* = .04) and extracranial disease progression (HR, 1.49; 95% CI, 1.01-2.19; *P* = .04) was associated with significantly shorter OS, while surgery for BrM (HR, 0.46; 95% CI, 0.25-0.87; p = 0.02) was associated with significantly longer OS (**Table 3**). The median OS for patients with endocrine-resistant disease was significantly shorter than that of patients with endocrine-sensitive disease, with a median survival of 9 months (95% CI, 6.5-12.1) as compared with 24.5 months (95% CI, 11.4-64.6), respectively (*P* = .002, **Figure 3**).

**Table 3.** Factors associated with overall survival in patients with HR+/HER2– BrM

	HR (95% CI)	*P* value
**Univariate**		
Breast cancer histology		
Ductal (ref)	1	
Lobular	0.912 (0.399-2.084)	.8276
Mixed	1.132 (0.55-2.333)	.7359
Age at MBC	1.141 (0.991-1.314)	.0663
Months from MBC to BrM (ln) × Chemotherapy in MBC prior to BrM	1.107 (1.012-1.211)	.0267
No (ref)	1	
Yes	1.723 (1.21-2.455)	.0026
BrM line 1		
Chemo	1	
Other sx tx	0.89 (0.602-1.316)	.5597
None	3.103 (1.887-5.102)	<.0001
Fulvestrant at BrM		
No (ref)	1	
Yes	0.282 (0.123-0.645)	.0027
Neurological symptoms at BrM diagnosis		
No symptoms (ref)	1	
Symptoms	1.205 (0.803-1.808)	.3674
EC progression at BrM diagnosis		
Stable (ref)	1	
Progressing	1.699 (1.178-2.45)	.0046
LMD		
No LMD (ref)	1	
LMD	1.195 (0.827-1.728)	.3427
Surgery for BrM		
No (ref)	1	
Yes	0.385 (0.214-0.693)	.0015
SRS only		
No (ref)	1	
Yes	0.942 (0.61-1.455)	.7865
**Multivariate**		
Chemotherapy in MBC prior to BrM		
No (ref)	1	
Yes	1.452 (1.013-2.083)	.0426
EC progression at BrM diagnosis		
Stable (ref)	1	
Progressing	1.487 (1.011-2.187)	.0439
Surgery for BrM		
No (ref)	1	
Yes	0.464 (0.247-0.872)	.017

## Discussion

In this single-centre retrospective cohort study, the great majority (78.4%) of patients with HR+/HER2– mBC and BrM had endocrine-resistant disease with a median survival of only 9 months. To our knowledge, our study is the first to demonstrate that among patients with mBC and BrM, those with endocrine-resistant HR+/HER2– disease have a similar median OS as compared to patients with metastatic triple negative breast cancer.^7^^,^^8^ Of the 13.7% of patients with endocrine-sensitive disease at the time of BrM diagnosis, a remarkably longer median OS of ∼2 years was observed. In addition, an unexpectedly high proportion (28.4%) of patients with HR+/HER2– mBC and BrM ultimately developed LMD, after which the median OS was only 5.3 months (95% CI, 3.4-9.0).

We found that a substantial proportion (∼19%) of patients were diagnosed with BrM concurrently with extracranial metastases, which is higher than the historically reported incidence of 6.1% from population analyses of SEER data.^19^ More recently, a meta-analysis of retrospective studies of brain imaging for breast cancer patients with symptomatic BrM reported a cumulative BrM incidence of 15% in patients with HR+/HER2– mBC,^20^ which may be attributed to improved efficacy of systemic therapies that improve extracranial disease control and survival.^20^^,^^21^ The fact that 23% of patients with HR+/HER2– breast cancer whose disease progressed on at least 1 line of endocrine-based therapy were found to have BrM at the time of either a baseline and/or 6-month brain MRI raises awareness about a previously unrecognized need for CNS-active systemic therapies for this patient population.^6^

We demonstrate that most patients with HR+/HER2– mBC develop BrM after a median of 2 lines of systemic therapy, and an average of 17.8 months from the time of initiating ET in the metastatic setting. For those already receiving chemotherapy, the average time from the start of cytotoxic therapy to the development of BrM was 11.5 months. This study suggests that endocrine-based approaches are unlikely to be effective for most patients with HR+/HER2– mBC and BrM due to endocrine resistance. However, 13.7% of patients in our cohort developed BrM in the setting of endocrine-sensitive disease with a median OS of ∼2 years, suggesting some patients have potential to benefit from endocrine-based strategies, findings which are supported by others.^22^

Despite being the mainstay treatment for HR+/HER2– mBC, the intracranial efficacy of ET is poorly understood. Unfortunately, we are not able to meaningfully comment on efficacy of ET plus CDK4/6 inhibition in our study (only 12.3% received this treatment combination). Single agent ET is unlikely to be effective in most patients given a very high incidence of endocrine resistant disease at the time of BrM diagnosis.^23^ Nevertheless, in select cases, aromatase inhibitors theoretically have potential to be effective for treating BrM as they can lower both serum and CSF concentrations of estradiol throughout the body; however, existing studies for the CNS activity of AIs in breast cancer BrM are ­limited.^23–27^ The efficacy of other targeted treatments such as imlunestrant plus abemaciclib^28^ and PI3K/AKT/mTOR inhibitors^10^^,^  ^14^ have demonstrated promising prolongation of overall PFS, however, their CNS-specific efficacy is not yet well established.

It is increasingly understood that systemic therapies can have CNS penetration and BrM efficacy given intrinsic disruption of the blood-brain barrier by the presence of BrM and/or associated local therapies. However, this remains a complex and evolving area of study with ongoing challenges in the limited delivery of pharmacotherapies to treat BrM across the BBB and blood-tumour barrier.^29^ Although clinical trial data for patients with active HR+/HER2– BrM is limited, some studies have suggested that systemic therapies with extra-cranial efficacy may have similar efficacy in the brain. For example, a retrospective cohort study examining 209 patients with all subtypes of mBC treated with capecitabine found a CNS objective response rate (ORR) of 38% in HR + HER2– mBC patients with a median PFS of 8.3 months.^30^ Similarly, methotrexate (MTX) has been shown to penetrate the BBB with a partial response rate of 28% in 29 patients with breast cancer.^31^ Capecitabine, etoposide, cisplatin, and high-dose MTX are listed as CNS-active options in NCCN guidelines.^32^ In addition, a recent phase II study that enrolled 46 patients with HR+/HER2– breast cancer and untreated or progressive BrM following radiation reported a CNS-ORR of 44% and 12-month OS rate of 74% with utidelone plus bevacizumab.^33^ However, this is currently not the standard of care in most jurisdictions and the results from this single trial should be interpreted with caution as they have not yet been replicated in larger studies.

Recently, several trials examined the activity of antibody drug conjugates (ADCs) among patients with mBC and BrM, although most are focused on those with HER2+ BC.^34^^,^^35^ The randomized phase 3 TROPiCS-02 trial evaluated sacituzumab govitecan in patients with endocrine- and chemotherapy-resistant HR+/HER2– mBC and included patients with stable BrM, although BrM-specific outcomes were not reported.^36^ The DESTINY-Breast04 trial, which evaluated the efficacy of T-DXd versus treatment of physicians’ choice chemotherapy among patients with HER2-low mBC included 32 (5.7%) patients with stable/previously treated BrM. The DEBBRAH study (NCT04420598) included a cohort of 4 patients with HER2-low MBC with progressing BrM after local therapy, and TUXEDO-3 (NCT05865990) is examining intra-cranial efficacy of HER3-DXd for patients with BrM from MBC (all subtypes) or non-small cell lung cancer. In addition, the intracranial efficacy of the Trop-2 directed ADC datopotamab deruxtecan (Dato-DXd) is being evaluated [NCT06176261 and NCT05866432].^37^

In our study, over a quarter (28.4%) of patients with HR+/HER2– mBC and BrM developed LMD, after which the median OS was only 5.3 months (95% CI, 3.4-9.0). This is in keeping with previous observational studies that have reported similarly poor prognosis for patients with HR+/HER2– LMD with a median OS of 3.7-8.4 months.^12^^,^  ^38–41^ Despite the low incidence of LMD occurring in approximately 5% of all patients with mBC, the HR+/HER2– subtype accounts for up to 66% of these cases^42^^,^^43^ with limited treatment options.^44^ In addition, we found a similar incidence of LMD in endocrine-sensitive versus endocrine-resistant tumors. This likely reflects the fact that endocrine responsiveness is not a primary determinant of leptomeningeal tropism. Rather, LMD risk in HR+/HER2− disease is likely more driven by intrinsic tumor characteristics (eg loss of cell adhesion), which are shared across endocrine sensitivity states and are particularly enriched in lobular histology.^45^ Given that endocrine therapies have limited and variable CSF penetration, there may be less clinical relevance of endocrine sensitivity for patients with LMD. Endocrine resistance mechanisms such as ESR1 mutations may drive systemic progression without conferring a selective advantage for leptomeningeal involvement or survival.^45^^,^^46^ Time-at-risk effects may also explain the observed similarity, as patients with endocrine-sensitive disease typically experience longer survival and prolonged exposure to risk of developing LMD, while those with endocrine-resistant disease experience earlier progression or death, resulting in comparable cumulative incidences of LMD.^47^

The median OS for patients with LMD that had endocrine-sensitive versus endocrine-resistant disease was not statistically different at 11.8 months (95% CI, 1.4-NA) versus 5.3 months (95% CI, 3.1-9), respectively. We suspect that survival outcomes are primarily driven by neurologic disease burden, performance status, and ability to deliver CNS-directed therapies (eg intrathecal therapy, systemic therapy, focal, or whole-brain radiotherapy), rather than prior endocrine sensitivity. We also hypothesize that endocrine sensitivity of extracranial disease may not accurately reflect tumor characteristics within the CNS or leptomeninges, where clonal selection, genomic evolution, and factors in the microenvironment can confer functional resistance independent of prior endocrine response.^48^^,^^49^ Given the disproportionate burden of LMD in patients with HR+/HER2 breast cancer, our study supports the need for high quality studies examining optimal treatment for this patient population.

Our study had several limitations, including those inherent to a retrospective design and the use of clinical treatment data as a surrogate for endocrine resistance. Mutations in the estrogen receptor (ER) alpha gene (ESR1) and/or PI3K mutational status were not available. It was also not possible to ascertain the cause of death (ie death due to CNS progression, extracranial progression, or non-cancer causes) in this cohort, and systemic treatment data were not available for 10% of patients. Additionally, our study has an inherent limitation of selection bias because all patients were seen in consultation for possible radiotherapy or received radiation therapy at some point during their treatment course. Most patients in our study were treated with WBRT in accordance with practice patterns at the time, although patients are now increasingly treated with SRS. Only approximately 12% of patients were treated with CDK4/6i during the timeframe of our study. More research with larger sample sizes and longer duration of follow-up are required to better understand the impact of CDK4/6i on the natural history and endocrine sensitivity of HR+/HER2– breast cancer BrM. Another limitation is that biomarker status of BrM tissue was not available in most cases because surgery is not routinely performed for BrM; this is relevant because a discordance in ER, PR and/or HER2 status between the ­primary tumour and BrM can occur.^50^^,^^51^ The diagnosis of LMD was based on clinical symptoms and local radiologic assessment, and imaging studies were not subject to centralized review. Further, not all patients had a brain MRI; 2.4% had a CT due to an inability to perform an MRI, largely as a result of claustrophobia. Lastly, this study was conducted at a ­single tertiary academic center and may have limited generalizability to the broader patient population. We acknowledge that the majority of patients in this study had endocrine-resistant disease, with only a small number having endocrine-sensitive disease. Hence, our findings require validation.

## Conclusion

A high proportion of patients with HR+/HER2– breast cancer who develop BrM have endocrine-resistant disease, poor survival outcomes and an unexpectedly high incidence (28%) of LMD. Patients with endocrine-sensitive disease at the time of BrM diagnosis have a prolonged survival of ∼2 years, suggesting possible benefit from endocrine-based approaches in select patient populations. More research is required to understand genomic drivers of BrM in patients with HR+/HER2– mBC and potential targets for treatment.

## Data Availability

De-identified data generated during and/or analysed during the current study are available from the corresponding author on reasonable request.
